# Skeletal Metastasis as Detected by 18F-FDG PET with Negative CT of the PET/CT: Frequency and Impact on Cancer Staging and/or Management

**DOI:** 10.3389/fonc.2016.00208

**Published:** 2016-10-10

**Authors:** Fatma Ahmed, Razi Muzaffar, Hermina Fernandes, Yifan Tu, Batool Albalooshi, Medhat M. Osman

**Affiliations:** ^1^Saint Louis University, Saint Louis, MO, USA; ^2^Dubai Hospital, Dubai, United Arab Emirates

**Keywords:** 18F-FDG PET/CT, skeletal metastasis, PET-positive, CT-negative

## Abstract

**Objectives:**

The aim of our study is to assess the frequency of detection of PET-positive computed tomography (CT)-negative skeletal metastases (SM) and determine the impact of such detection on staging and/or management in patients who had FDG PET/CT as part of the cancer work-up.

**Methods:**

We retrospectively reviewed 2000 18F-FDG PET/CT scans of known cancer patients. A log was kept to record cases of suspected SM with or without bone changes from the low-dose non-contrast CT. The presence or absence of SM was evaluated based on available pathological and clinical data. The impact of detection of such lesions on cancer staging and/or management was evaluated by a board certified oncologist.

**Results:**

Of the 2000 cases, 18F-FDG PET/CT suggested SM in 146/2000 (7.3%). Of those 146 cases, 105 (72%) were positive on both PET and CT. The remaining 41 (28%) had PET-positive CT-negative bone lesions. SM was confirmed in 36/41 (88%) PET-positive/CT-negative cases. This was based on biopsy, imaging, or clinical follow-up. The detection of PET-positive CT-negative SM did not change staging or management in 7/36 (19.4%). However, staging and/or management was affected in 29/36 (80.6%).

**Conclusion:**

SM is not uncommon in 18F-FDG PET/CT, as it accounts for 146/2000 (7.3%) of cases. PET demonstrated FDG-avid SM without a CT abnormality in at least 36/146 (25%). Patients staging and/or management changed in 29/36 (80.5%). We concluded that 18F-FDG PET is sensitive in the detection of SM with significant impact on staging and/or management.

## Introduction

Early diagnosis of cancer remains to be of paramount importance to maximize a patient’s long-term survival and reduce various neurological, hematological, and orthopedic complications that may arise. Skeletal metastasis (SM) is a frequently encountered and important complication of cancer which can lead to intolerable pain. Bone metastases are strongly associated with increased risk of death and increased risk of skeletal-related events (SREs) (1, 2). SREs are associated with increased morbidity, increased treatment costs, and reduced quality of life (3, 4). Skeletal metastases occur in more than 50% of malignant tumors and up to 70% of breast cancer patients (5, 6). Patients with renal cell carcinoma (RCC) and SM demonstrated unfavorable prognosis in which more than 50% of patients die within the first year of diagnosis of SM (7–10). The detection of SM might affect cancer staging and follow-up, as it can affect the treatment plan. This is particularly true in breast, prostate, and lung cancer patients, which are the most common malignancies in the United States, with a profound impact on patient prognosis (11–15). Whole-body 18F-FDG PET/CT allows for the metabolic, anatomic, and morphological characterization of suspected bone lesions (16, 17). Studies have shown that the higher sensitivity and specificity of 18F-FDG PET/CT in the detection of metastasis as well as the impact on tumor staging makes it superior to other imaging modalities (18, 19). Although computed tomography (CT) is a standard imaging modality in cancer imaging, it may fail to detect metastasis, which is confined to the bone marrow or to the cortex without visible bone destruction (20, 21). The purpose of our study is to retrospectively assess the frequency of detection of PET-positive/CT-negative SM and determine the impact of its detection on cancer staging and/or management in patients who had 18F-FDG PET/CT as part of their cancer work-up.

## Materials and Methods

### Patient Selection

After obtaining Saint Louis University Institutional Review Board (IRB) approval, we retrospectively reviewed 2000 consecutive 18F-FDG PET/CT scans of known cancer patients. The vast majority of patients had top of the head to bottom of the feet 18F-FDG PET/CT, as it is the standard of care in our institution. A log was kept for cases with suspected SM. PET-positive cases were further evaluated for the presence of bone window changes in the CT. The presence or absence of SM in PET-positive/CT-negative cases was determined based on all available pathological and clinical data. The impact of detection of SM on cancer staging and/or management was evaluated by a board certified oncologist.

### PET/CT Scanning

18F-FDG PET/CT scans were acquired using PET/CT scanner (Gemini TF; Philips Medical Systems) with an axial co-scan range of 193 cm. Per institutional protocol, all patients were instructed to fast at least 4 h prior to receiving the radiopharmaceutical injection. Blood glucose level was <200 mg/dl in all patients. On the day of the exam, intravenous injection of 5.18 MBq/kg (0.14 mCi/kg) of F18-FDG was administrated. For the uptake phase, patients sat in a quiet room without talking for about 60 min.

### CT Scanning

The CT component of the PET/CT scanner has 64 multidetector helical CT with a gantry port of 70 cm. The parameters of CT detectors were set as follow for 20–21 bed acquisitions: 120–140 kV and 33–100 mAs (based on body mass index), 0.5 s per CT rotation, pitch of 0.9, and 512 × 512 matrix data were used for image fusion and the generation of the CT transmission map. The CT images were obtained without oral or IV contrast administration according to the standard PET/CT protocol at our institution.

### PET Scanning and Image Processing

The PET component of the PET/CT scanner is composed of lutetium–yttrium oxyorthosilicate (LYSO)-based crystal. Emission scans were acquired at 1–2 min per bed position. The FOV was from the top-of-head to the bottom-of-feet in vast majority of patients. The three-dimensional (3D) whole-body WB acquisition parameters were 128 × 128 matrix and 18 cm FOV with a 50% overlap. Processing used the 3D Row Action Maximum Likelihood Algorithm (RAMLA) method. Total scan time per patient was approximately 20–45 min.

### Image Analysis

PET/CT images were retrospectively evaluated on the Gemini TF extended brilliance workstation (EBW) by a board certified nuclear medicine physician and a fellowship trained radiologist. Cases were categorized into two groups: the first group was cases with PET-positive and CT-positive bone lesions, in which the CT of the PET/CT demonstrated morphological changes whether lytic, sclerotic, or mixed. The second group was PET-positive and CT-negative for bone lesions, in which the CT of the PET/CT failed to demonstrate definite morphological bone or bone marrow changes. No minimum size, number, or standardized uptake value criterion was assigned to the SM for inclusion into the study. A thorough review of the medical records, including clinical notes, pathology reports, and radiology reports, was performed by a board certified oncologist to confirm the diagnosis of SM and convey the impact of its detection on cancer staging and/or management.

## Results

**Figure 1:**
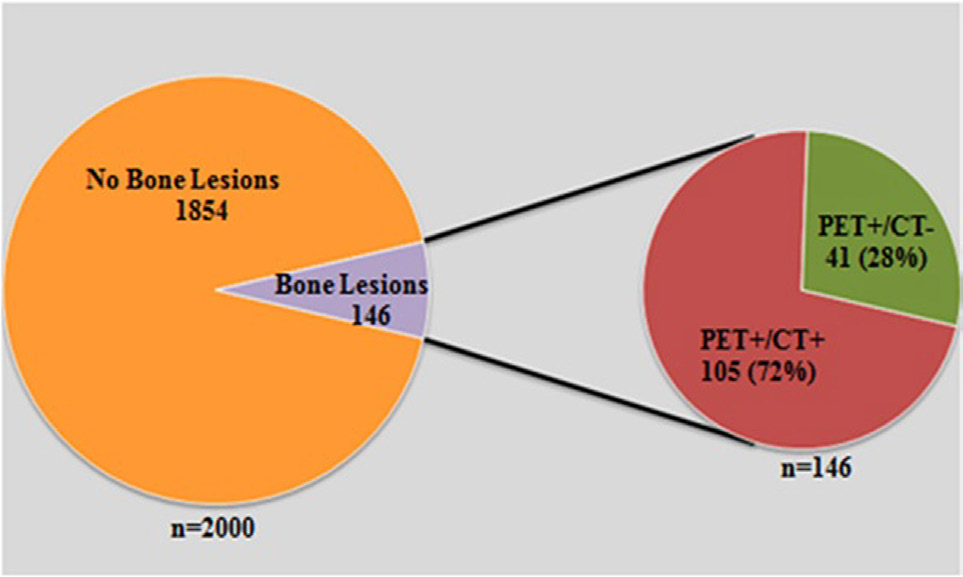
**A graph demonstrated total number of scans that were reviewed 2000**. Of those, 146 had suspected SM by PET and CT. However, 41 cases were only positive by PET.

**Figure 2:**
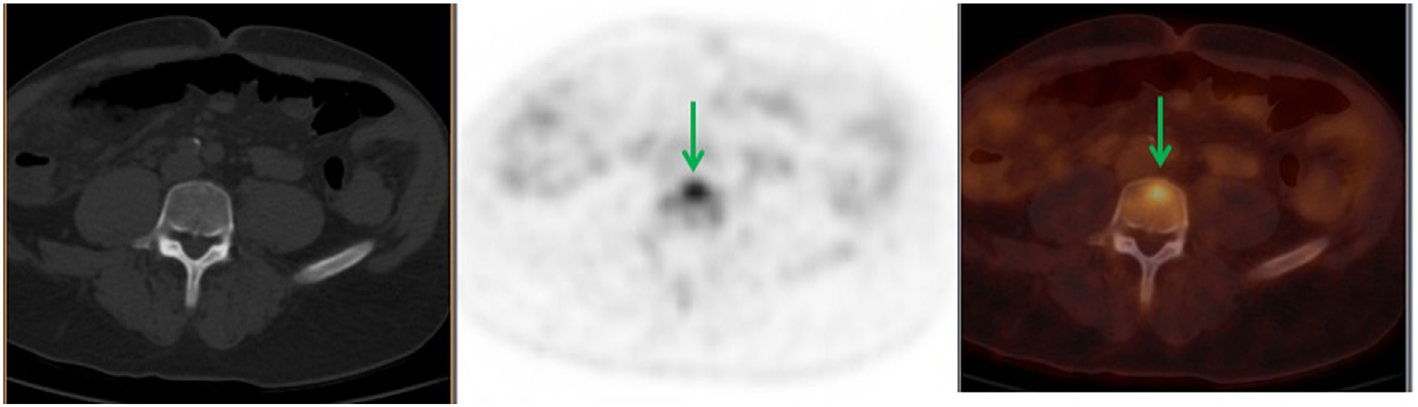
**A 56-year-old male diagnosed with stage IIIC adenocarcinoma of the ascending colon status post right hemicolectomy**. Follow-up, 18F-FDG PET demonstrated L5 FDG-avid lesion (green arrows) with no corresponding bone changes in the CT. This finding upstaged his cancer to stage IV. Patient received XRT and offered hospice.

**Figure 3:**
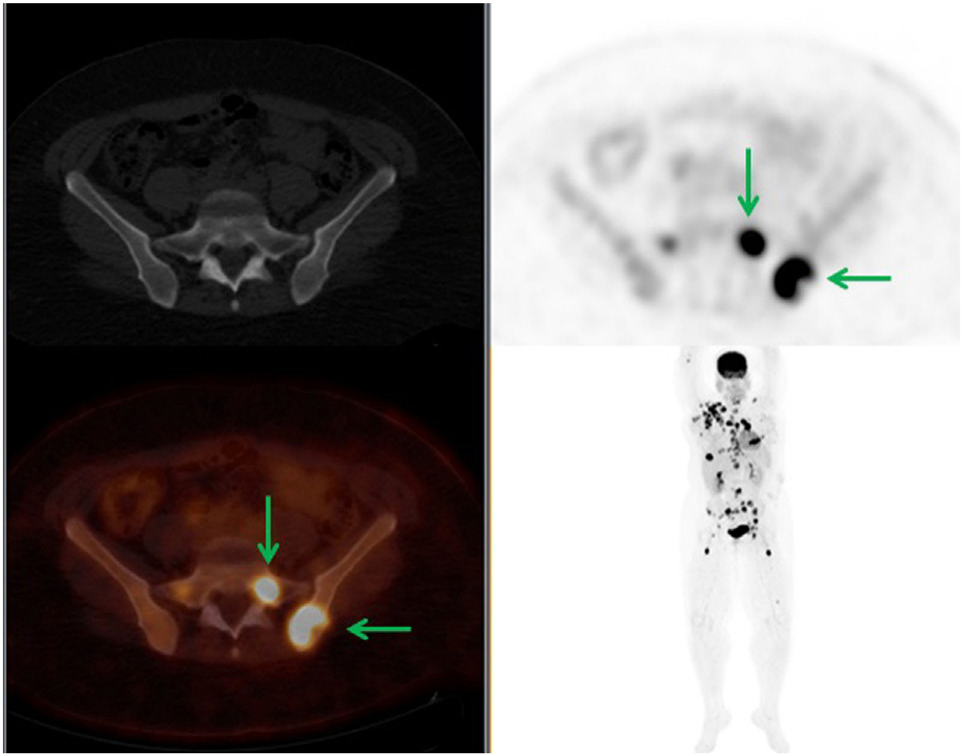
**A 34-year-old female diagnosed with right breast cancer**. 18F-FDG PET demonstrated widespread disease with PET-positive CT-negative SM. The FDG-avid left iliac bone and sacral lesions demonstrated no bone changes in the CT (green arrows). In this case, there was no change in staging; however, BMA was offered.

**Figure 4:**
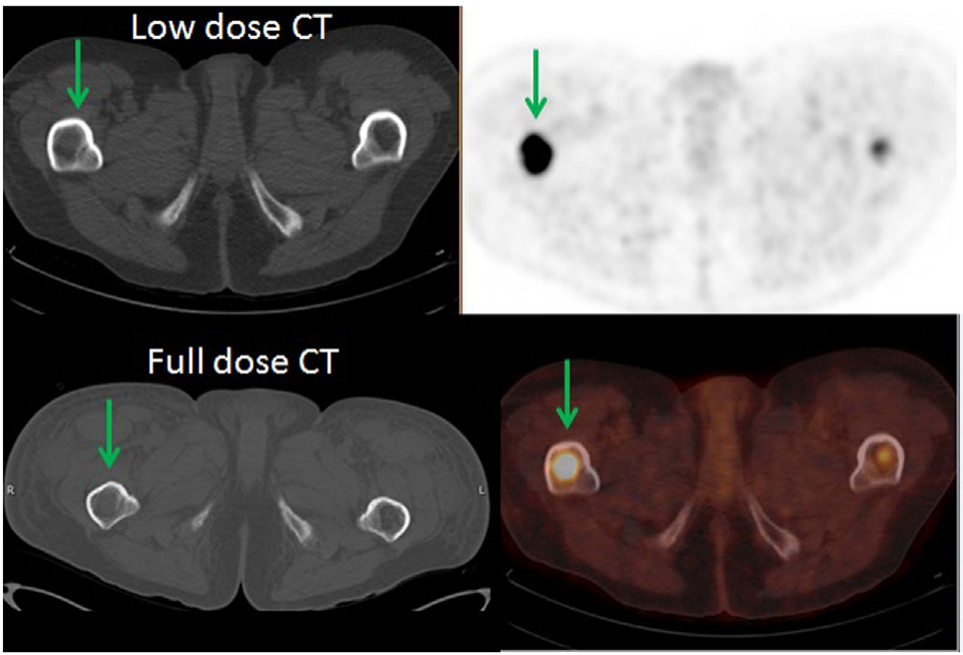
**A 53-year-old male diagnosed with diffuse large B-cell lymphoma with multiple FDG-avid lesions above and below the diaphragm**. There were PET-positive CT-negative femoral lesions (green arrows). Patient had a full dose CT which was negative for SM. There was no change in staging but XRT and BMA were offered.

**Figure 5:**
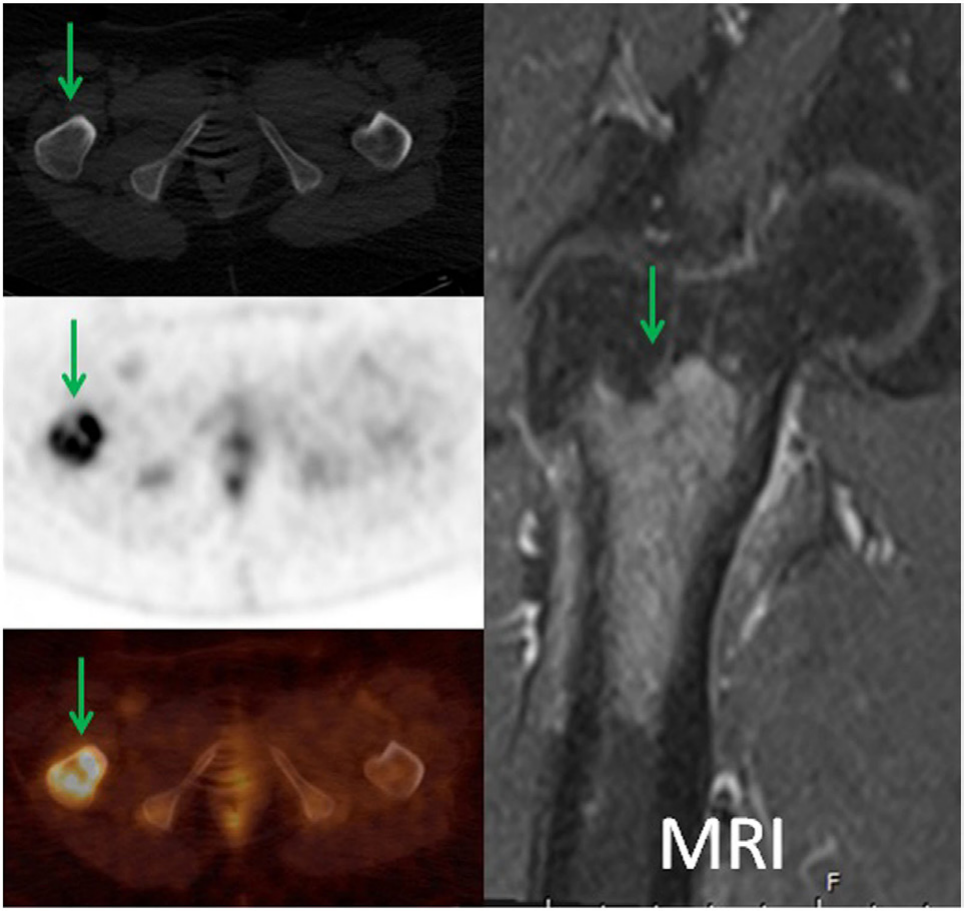
**A 57-year-old female diagnosed with chronic myelogenous leukemia status post allogenic bone marrow stem cell transplant and widespread disease**. 18F-FDG PET demonstrated intense focal uptake in the intertrochanteric region of the proximal right femur with no corresponding bone changes in the CT (green arrows). The patient had MRI with contrast which demonstrated marrow replacing lesion. Patient received XRT, BMA, and had orthopedic fixation. In addition, 18F-FDG PET directed the biopsy site for this patient.

Of the 2000 cases, 18F-FDG PET/CT suggested bone metastases in 146/2000 (7.3%). In those 146 cases, 105 (72%) were positive on both PET and CT. The remaining 41 (28%) had PET-positive/CT-negative bone lesions (Figure 1). SM was confirmed in 36/41 (88%) PET-positive/CT-negative cases. This was based on biopsy, imaging, or clinical follow-up. The primary malignancies in these 36 patients included: breast cancer (*n* = 8), lymphoma (*n* = 7), lung cancer (*n* = 6), melanoma (*n* = 4), leukemia (*n* = 3), multiple myeloma (*n* = 2), gastrointestinal cancers (*n* = 2), and others (*n* = 4). Of the remaining 5/41 (12%), SM was not confirmed since one patient had a negative bone biopsy and the other four patients had no follow-up. The impact of detection of PET-positive CT-negative SM on staging and/or management was determined by a board certified oncologist. The detection of PET-positive/CT-negative SM did not change staging or management in 7/36 (19.4%), because these patients had widespread disease. In total, staging and/or management was affected in 29/36 (80.6%). This is further divided into patients with a change in staging and management in 4/36 (11%), change in staging only in 1/36 (3%), and change in management only in 24/36 (67%) (Figures 2 and 3). The change in management was in the form of addition or change in chemotherapy in 16 patients, addition of bone-modifying agents (BMA) in 12 patients, 11 patients received radiotherapy (XRT) to their bone metastasis, 2 patients had orthopedic fixation, and hospice was offered for 4 patients (Figures 4 and 5; Table 1).

**Table 1:** **Patients in whom the detection of PET-positive CT-negative SM change staging and/or management**.

Patient no.	Age (years)	Sex	Type of primary tumor	Change in stage	Change in management
1	59	M	Esophageal	No	Yes
2	86	F	Breast	No	Yes
3	49	F	Melanoma	No	Yes
4	81	M	Head and neck	No	Yes
5	57	M	Lung	No	Yes
6	30	M	Lymphoma	No	Yes
7	57	F	Lung	Yes III → IV	Yes
8	63	M	GI	YES IIIC → IV	Yes
9	48	M	Melanoma	No	Yes
10	62	F	breast	No	Yes
11	74	F	Multiple myleoma	No	Yes
12	61	M	Lymphoma	No	Yes
13	36	F	Breast	No	Yes
14	52	F	Germ cell	No	Yes
15	73	F	Breast	No	Yes
16	71	F	Lymphoma	No	Yes
17	62	M	Lung	No	Yes
18	79	M	Lung	No	Yes
19	68	M	GI	No	Yes
20	55	M	Lymphoma	No	Yes
21	66	F	Lung	Yes III → IV	No
22	69	F	Breast	No	Yes
23	80	F	Breast	NO	Yes
24	53	F	Lung	Yes III → IV	Yes
25	58	F	Leukemia	NO	Yes
26	57	F	Lung	No	Yes
27	59	M	GI	Yes IIIC → IV	Yes
28	70	M	Melanoma	No	Yes
29	77	M	MM	No	Yes

## Discussion

Skeletal metastasis is a serious complication of malignant tumors with a significant impact on quality of life. It causes several serious complications that have emerged as a collection of defined SREs including pathological fractures, spinal cord compression, the need for surgery or radiation for a symptomatic bone lesion, and hypercalcemia of malignancy (21, 22). Although the exact incidence of SM in the broad population is unknown, it is estimated that skeletal involvement is present in more than half of those deaths resulting from advanced cancer (23).

18F-FDG PET/CT demonstrated higher sensitivity and specificity in the detection of SM. This additional information for tumor staging makes it superior to other imaging modalities (18, 19). In a study done by Nakai et al., they described invisible SM which was not visualized by CT but demonstrated increased FDG uptake by PET. They concluded that 18F-FDG PET/CT has higher sensitivity than CT in the detection of microscopic early SM and attributed these findings to high glucose metabolism by tumor cells (20). In a retrospective study, Uchida et al. evaluated the efficacy of 18F-FDG PET/CT in the detection of suspected bone marrow metastases in the spine and compared it to bone scan. In their study, the CT of the 18F-FDG PET/CT was able to characterize only 31–32% of bone marrow metastases using all available resources, whereas 68–69% went undiagnosed using CT (21). Chang et al., in a meta-analysis, described increased sensitivity and specificity of 18F-FDG PET/CT in the detection of bone metastases from lung cancer compared with conventional bone scan. They reported a sensitivity of 93% and specificity of 95% by PET compared with sensitivity of 87% and specificity of 82% by bone scan (19). In our study, we compared the sensitivity of PET to low-dose CT of the PET/CT in the detection of SM.

Our study demonstrated that PET can show an FDG-avid SM without a CT abnormality in at least 36/146 (25%) of cases. We related the discrepancy between PET and CT in the detection of SM to the mechanism by which FDG is accumulated into the SM by viable and metabolically active tumor cells, which is visible by PET before the detection of anatomic changes in the CT. Detection of such lesions changed staging and/or management in 29/36 (80.6%) of patients who needed special treatment to SM. Although we did not evaluate the effect of the detection of SM on overall survival (OS), research studies found that detection of SM may have an impact on prognosis and OS in some cancers. This is particularly true in breast cancer patients with bone marrow metastases and is even worse if it is complicated by pathological fracture (24). Demir et al. reported that the median survival time after the diagnosis of bone marrow metastases was 6.43 months which was significantly prolonged after systemic therapy (17.3 versus 0.93 months, 95% CI; *p* < 0.001) (25). These findings are similar to our results, in which two patients demonstrated bone marrow metastases detect by PET and both passed away a few months after the diagnosis of marrow metastases. In addition, 18F-FDG PET/CT aided in diagnosis of SM by directing the site of bone marrow biopsy as in the patient in Figure 5 in which the bone marrow biopsy was negative at the iliac region and positive at the right femur. We did not evaluate the effect of detection of PET-positive, CT-negative SM in prostate cancer patients. The growing use of (18)F sodium fluoride [(18)F NaF] (PET/CT) in the detection of SM in prostate cancer patients, especially those with rising PSA level and negative bone scan, resulted in under representation of prostate cancer patients in our FDG PET/CT scans. Studies have shown that 18F NaF PET/CT is more sensitive than both 99mTc-MDP bone scintigraphy and 18F-FDG PET/CT in the detection and differentiation of SM from degenerative changes in prostate cancer patients (26–29).

Our study is not without limitations. We compared the sensitivity of PET to the low-dose CT of the PET/CT because it is the standard of care in our institution. Indeed, most of the patients did not have full dose diagnostic CT available to compare to the PET/CT. However, it is not likely that the diagnostic accuracy of contrast CT would have been significantly better than on low-dose non-contrast CT. Two of the patients had full dose diagnostic CT done within a month from the 18F-FDG PET/CT and neither demonstrated corresponding changes in CT bone window (Figures 4 and 5). Another limitation is that our study is based solely on the findings of PET/CT without correlation with other imaging modalities. This is related to the retrospective nature of our study that made us rely more on clinical and/or pathological confirmation of SM. Finally, the relatively small number of PET-positive/CT-negative bone metastases did not allow evaluation of the possible impact on staging.

## Conclusion

In our study, SM was not uncommon in 18F-FDG PET/CT, as it accounts for 146/2000 (7.3%) of our cases. PET demonstrated FDG-avid SM without a CT abnormality in at least 36/146 (25%) which resulted in a staging and/or management change in 29/36 (80.5%). We concluded that 18F-FDG PET may be sensitive in the detection of SM with a significant impact on patient staging and/or management.

## Author Contributions

FA contributed in data gathering and writing of the manuscript. RM and MO contributed in image analysis and writing of the manuscript. HF and YT contributed in writing of the manuscript and detection of the impact of PET-positive CT-negative SM on management and/or staging. BA and MO reviewed the manuscript and edited the discussion.

## Conflict of Interest Statement

The authors declare that the research was conducted in the absence of any commercial or financial relationships that could be construed as a potential conflict of interest.
